# Peptide Agonists of Vasopressin V2 Receptor Reduce Expression of Neuroendocrine Markers and Tumor Growth in Human Lung and Prostate Tumor Cells

**DOI:** 10.3389/fonc.2017.00011

**Published:** 2017-01-30

**Authors:** Marina Pifano, Juan Garona, Carla S. Capobianco, Nazareno Gonzalez, Daniel F. Alonso, Giselle V. Ripoll

**Affiliations:** ^1^Laboratory of Molecular Oncology, Quilmes National University, Bernal, Buenos Aires, Argentina

**Keywords:** neuroendocrine tumors, vasopressin analogs, V2r, antitumor, SCLC, prostate cancer

## Abstract

Neuroendocrine tumors (NETs) comprise a heterogeneous group of malignancies that express neuropeptides as synaptophysin, chromogranin A (CgA), and specific neuronal enolase (NSE), among others. Vasopressin (AVP) is a neuropeptide with an endocrine, paracrine, and autocrine effect in normal and pathological tissues. AVP receptors are present in human lung, breast, pancreatic, colorectal, and gastrointestinal tumors. While AVP V1 receptors are associated with stimulation of cellular proliferation, AVP V2 receptor (V2r) is related to antiproliferative effects. Desmopressin (dDAVP) is a synthetic analog of AVP that acts as a selective agonist for the V2r, which shows antitumor properties in breast and colorectal cancer models. Recently, we developed a derivative of dDAVP named [V^4^Q^5^]dDAVP, which presents higher antitumor effects in a breast cancer model compared to the parental compound. The goal of present work was to explore the antitumor properties of the V2r agonist dDAVP and its novel analog [V^4^Q^5^]dDAVP on aggressive human lung (NCI-H82) and prostate cancer (PC-3) cell lines with neuroendocrine (NE) characteristics. We study the presence of specific NE markers (CgA and NSE) and V2r expression in NCI-H82 and PC-3. Both cell lines express high levels of NE markers NSE and CgA but then incubation with dDAVP diminished expression levels of both markers. DDAVP and [V^4^Q^5^]dDAVP significantly reduced proliferation, doubling time, and migration in both tumor cell cultures. [V^4^Q^5^]dDAVP analog showed a higher cytostatic effect than dDAVP, on cellular proliferation in the NCI-H82 cell line. Silencing of V2r using small interfering RNA significantly attenuated the inhibitory effects of [V^4^Q^5^]dDAVP on NCI-H82 cell proliferation. We, preliminarily, explored the *in vivo* effect of dDAVP and [V^4^Q^5^]dDAVP on NCI-H82 small cell lung cancer xenografts. Treated tumors (0.3 μg kg^−1^, thrice a week) grew slower in comparison to vehicle-treated animals. In this work, we demonstrated that the specific agonists of V2r, dDAVP, and [V^4^Q^5^]dDAVP displays antitumor capacity on different human models of lung and prostate cancers with NE features, showing their potential therapeutic benefits in the treatment of these aggressive tumors.

## Introduction

Neuroendocrine tumors (NETs) comprise a diverse group of malignancies that present a broad range of morphological, functional, and behavioral characteristics. The concept of neuroendocrine (NE) differentiation can be defıned as the secretion of bioactive substances by the neoplastic cells (1).

Neuroendocrine tumors growth is mainly regulated by autocrine and paracrine signaling through neuropeptides, peptide hormones, and bioamines with defined physiologic actions, and it has been widely reported in lung, gastric, colorectal, pancreatic, and prostatic cancer (2, 3). These signaling pathways depend on the expression of specific receptors on tumor cells. Among others, proteins expressed by these tumors are the synaptophysin, the chromogranin A (CgA), and the specific neuronal enolase (NSE) (4, 5). CgA is the most widely used biomarker for detecting NE differentiation in prostate cancer either at tissue level or in general circulation (6). NSE is currently the most reliable tumor marker in diagnosis, prognosis, and follow-up of small cell lung cancer (SCLC) (7–10).

Somastostatin (SST) is a neuropeptide with paracrine activity secreted by intestinal, lung, pancreas, and different nerve cells. It acts as a strong secretion inhibitor of several hormones as insulin, gastrin, and some growth factors. It also inhibits the proliferation of both normal and tumor cells. Its biological activity is exerted through binding to five different G-protein-coupled receptors (GPCRs) (11). Several analogs of somatostatin have been developed showing a potent biological activity with an enhanced stability in comparison to the parental peptide. It has been demonstrated that somatostatin and its analogs display antiangiogenic and antiproliferative activity on tumor cells both *in vitro* and *in vivo*, highlighting that some neuropeptides and specially its synthetic derivatives could be potentially used as antitumor agents. In particular, SST analogs (SSA) are used frequently to control hormone-related symptoms in NE cancer patients while their anti-neoplastic activity seems to result prevalently in tumor stabilization (12).

Vasopressin (AVP also called antidiuretic hormone) is a neuropeptide hormone synthesized primarily in the hypothalamus. Mainly, it has an endocrine effect but it is also secreted locally by many normal and pathological tissues exerting autocrine and paracrine activities (13). The effects of AVP are mediated by three GPCRs. These receptors are AVP type 1 receptor (V1ar, V1br also called V3r) and AVP type 2 receptor (V2r) and have numerous actions centrally and in the periphery (14, 15). The V1ar is typically expressed in vascular smooth muscle cells, where it mediates smooth muscle contraction and is also present in brain, adrenal cortex, adipose tissue, and hepatocytes. The V1br is mainly expressed in the anterior pituitary gland, adrenal medulla, islet cells of Langerhans, and white adipose tissue regulating the hypothalamic–pituitary–adrenal axis. V2r is expressed in the collecting ducts of the kidney and in alveolar vascular endothelium epithelial cells, stimulating water absorption and release of hemostatic factors from micro vasculature (16).

Interestingly, there is evidence of the expression of AVP and their receptors in SCLC (17, 18), breast (19, 20), pancreatic and colorectal cancer, and human gastrointestinal tumors (1). Moreover, syndrome of inappropriate antidiuretic hormone secretion was reported in patients with prostate cancer and SCLC (21, 22).

While V1 receptors are associated with the stimulation of cellular proliferation, V2r is related to antiproliferative effects (23). Desmopressin (dDAVP) is a synthetic peptide analog of AVP that acts as a selective agonist for the V2r. DDAVP differs from AVP by deamination of cystein in position 1, which prolongs its half-life; and substitution of l-arginine by d-arginine in position 8, which reduces the pressor effect and confers selectivity for V2r (24).

In our laboratory, we reported for the first time that dDAVP inhibits the dissemination of aggressive breast cancer cells (25) and colon carcinoma cells (26). We, further, demonstrated that peri-operative dDAVP treatment dramatically reduces lymph node and lung metastasis in a mouse model of mammary tumor manipulation and surgical excision (27).

Besides its antimetastatic properties, dDAVP has cytostatic properties and induces a tumor-mediated production of angiostatin, a potent antiangiogenic effector (28, 29). Thereby dDAVP seems to induce a dual, angiostatic and antimetastatic, effect, disrupting the crosstalk of cancer cells and endothelial cells during tumor progression (30).

Desmopressin was rationally modified in our laboratory to improve its biological activity. Parental peptide dDAVP was substituted in positions 4 and 5, and the novel analog [V^4^Q^5^]dDAVP ([4-valine 5-glutamine] desmopressin) was synthesized and evaluated. Amino acid positions 4 and 5 belong to the conformational peptide loop which has a key role in ligand–receptor interaction and antitumor activity (31). [V^4^Q^5^]dDAVP exhibited a significantly higher cytostatic effect against breast cancer cells compared to the parental compound, inhibited angiogenesis in endothelial cell cultures, and also reduced tumor growth and angiogenesis in aggressive human breast cancer xenograft (32). Additionally, [V^4^Q^5^]dDAVP, in combination with cytotoxic agents, resulted in a cooperative inhibition of breast cancer growth and metastatic progression in comparison to single-agent therapy (33).

Considering the evidence that supports the autocrine and paracrine involvement of neuropeptides in the regulation of tumor growth in cancers, such as lung and prostate, the present work explores the antitumor properties of the V2r agonist dDAVP and its novel analog [V^4^Q^5^]dDAVP on aggressive tumor cells with NE characteristics.

## Materials and Methods

### Cell Culture

Human SCLC cell line NCI-H82 (HTB-175), human non-small lung cancer cell line NCI-H125 (CRL-5801), and human prostate adenocarcinoma cell line PC-3 (CRL-1435) were obtained from the American Type Culture Collection. Tumor cells were grown in RPMI-1640 medium (Gibco, Rockville, MD, USA) supplemented with 10% fetal bovine serum (FBS), 2 mg ml^−1^ glutamine, and 80 μg ml^−1^ gentamycin as floating aggregates or monolayer culture, at 37°C in a humidified atmosphere of 5% CO_2_. Cells were harvested using a trypsin and EDTA solution (Gibco, Rockville, MD, USA) diluted in phosphate-buffered saline (PBS).

### Peptide Compounds

Desmopressin and its analog [V^4^Q^5^]dDAVP were purchased from American Peptide Company Inc. (CA, USA, current BACHEM).

### Immunofluorescence Detection of V2r

V2r expression was evaluated by immunofluorescence as described elsewhere (29). Briefly, NCI-H125 and PC-3 cancer cells were seeded on glass coverslips and fixed with 4% paraformaldehyde solution in PBS. NCI-H82 cells were subjected to centrifugation at 3,800 × *g* for 10 min. The supernatant fluids were drained off and cells were fixed with 4% paraformaldehyde solution in PBS. After incubation with blocking agent, cells were incubated with an anti-V2r primary antibody for 1 h at 37°C (Santa Cruz Biotechnology, Santa Cruz, CA, USA). Receptor-bound antibodies were detected with a secondary FITC-conjugated goat anti-rabbit IgG (Chemicon International, Temecula, CA, USA) and nuclei were labeled with DAPI using the Vectashield fluorescent mounting medium (Vector Laboratories, Peterborough, UK). Samples were examined using a TE-2000 fluorescence microscope (Nikon Inc., Tokyo, Japan). Cultures of MDA-MB-231 human breast carcinoma cells were used as a positive control of V2r expression (32). Negative controls consisted of omission of the primary antibody.

### Quantitative RT-PCR (qRT-PCR)

Total RNA of NCI-H82, NCI-H125, and PC-3 treated or not with dDAVP 1,000 nM overnight (ON) was purified from 1 × 10^6^ cells with Trizol according to the manufacturer’s protocol. RNA was reverse transcribed with SuperScript III first-Strand (Thermo Fisher Scientific Inc., USA) according to the manufacturer’s protocol. The following specific forward and reverse primers were used as described elsewhere (34): for NSE, 5-GAGACAAACAGCGTTACTTAG-30 and 50-AGCTGCCCCTGCCTTAC-3; for CgA, 5-GCGGTGGAAGAGCCATCAT-3 and 5-TCTGTGGCTTCACCACTTTTCTC-3; and for glyceraldehyde 3-phosphate dehydrogenase (GAPDH), 5-CATGGGTGTGAACCATGAGA-3 and 5-CAGTGATGGCATGGACTGTG-3 (35).

Quantitative RT-PCR was performed using SYBR Green PCR Master Mix (Thermo Fisher Scientific Inc., USA) and StepOne Real-Time PCR System (Applied Biosystems, Foster City, CA, USA). The following thermal cycling conditions were used: 48°C for 30 min, 95°C for 10 min, 40 cycles of 95°C for 15 s followed by 60°C for 60 s. Each sample was analyzed in triplicate and mean cycle threshold values (Ct) were used for further analysis. Ct values were normalized for GAPDH expression levels and normalized to control samples. Relative quantification values were calculated as 2^−δδCt^.

### Cell Proliferation Assay

Antiproliferative effect of AVP analogs was measured on rapidly growing tumor cells using MTT (3-(4,5-dimethylthiazol-2-yl)-2,5-diphenyltetrazolium bromide; Sigma-Aldrich, St. Louis, MO, USA) or MTS assay [3-(4, 5-dimethylthiazol-2-yl)-5-(3-carboxymethoxyphenyl)-2-(4-sulfophenyl)-2H-tetrazolium; Promega, Madison, WI, USA]. Briefly, depending on tumor cell line, cells were plated in 96-well flat bottom plates at a density of 3–5 × 10^3^ cell/well in 200 µl in RPMI-1640 supplemented with 10% FBS, allowed to attach ON, and then treated with dDAVP or [V^4^Q^5^]dDAVP (100–1,500 nM) or vehicle for 72 h. MTT or MTS reagent (20 µl) was added to each well and the plate was incubated at 37°C for 2–4 h. For MTT the absorbance of each well was measured in a microplate reader at 570 nm after solubilization using dimethyl sulfoxide. For MTS, absorbance was measured at 490 nm. The optical density of control cells was considered as 100% viability.

### Doubling Time

For doubling time calculation, cells were seeded at 3 × 10^4^ cells/well in 12-well plate in presence or not of dDAVP 1,000 nM. Viable cells were counted every 24 h for 72 h using trypan blue dye exclusion. Doubling time was calculated during the last 3 days of the exponential growth phase.

### Small Interfering RNA (siRNA)

To study the interaction of [V^4^Q^5^]dDAVP with V2r, we interfered the expression of the molecule. The sequences of siRNA for the sense and antisense strands were: for anti-V2r siRNA, sense 5′-GAGGAUGACGCUAGUGAUUTT-3′ and antisense 3′-TTCUCCUACUGCGAUCACUAA-5′; for control siRNA, sense 5′-CAGUCAGGAGGAUCCAAAGTT-3′ and antisense 3′-TTGUCAGUCCUCCUAGGUUUC-5′ (36). Synthetic oligonucleotides (Thermo Fisher Scientific Inc., USA) were annealed to form a short double-stranded RNA with a 3′-dithymidine overhang. On day 0, NCI-H82 and NCI-H125 cells were plated on 60 mm dishes, grown until approximately 50–60% confluency and then transfected with V2r siRNA or control siRNA using Lipofectamine 2000 (Thermo Fisher Scientific Inc., USA), following manufacturer’s instructions. After 4 days, cells were submitted to a second round of transfection under the same conditions. On day 3, 7.5 × 10^3^ cells were plated in 96-well plates and then treated with different concentrations of [V^4^Q^5^]dDAVP for 72 h.

### Clonogenic Assay

Cytostatic effects of dDAVP and [V^4^Q^5^]dDAVP on tumor cells were also examined at low-density cultures by the colony formation assay. 300 PC-3 cells/well were grown in complete medium in the presence of dDAVP or [V^4^Q^5^]dDAVP (100–1,500 nM). Complete medium with peptidic compounds was renewed after 72 h. Seven days after cell seeding, colonies of more than 30 cells were counted.

### Migration Transwell^®^ Assay

After on starvation, 3 × 10^5^ NCI-H82 cells or 3 × 10^3^ NCI-H125 cells were seeded into the Transwell^®^ inserts with 8 µm pore in 24-transwell cell culture dishes (Corning, NY, USA) in serum-free medium. The lower chamber was filled with medium containing 10% FBS as chemoattractant. After 24 h incubation with dDAVP or [V^4^Q^5^]dDAVP at a final concentration of 1,000 nM, stationary cells were removed from the upper surface of the membranes with a cotton swab. Cells that migrated to the lower surface were fixed and stained with crystal violet. Migrating cells in five randomly selected fields were counted and normalized to control.

### Wound Healing Migration Assay

Cell migration was measured using an *in vitro* wound healing assay as described by Segatori et al. (37). Briefly, “scratch” wounds were created by scraping confluent PC-3 monolayers with a sterile pipette tip. After 16 h incubation in RPMI 1640 supplemented with 10% FBS in the presence or absence of dDAVP or [V^4^Q^5^]dDAVP at a final concentration of 1,000 nM, cells were fixed and stained. Ten random micrographs per well were obtained and migration area was quantified using Image J. software (https://imagej.nih.gov/ij/). Wound-closure measurements were normalized to the maximum “scratch” area.

### Mice

Specific pathogen-free 8-week-old male athymic N:NIH(S)-nu mice were purchased from UNLP (Universidad Nacional de La Plata, Buenos Aires, Argentina) and kept, five to eight mice per cage, in our animal house facility at the National University of Quilmes. Food and water were provided *ad libitum*, and general health status of the animals was monitored daily. All protocols were approved by the National University of Quilmes institutional Animal Care Committee.

### Tumor Progression

To generate lung cancer xenografts, 1 × 10^5^ NCI-H82 cells in 300 µl containing serum-free RPMI-1640 and Matrigel^®^ (1:1 volume ratio) were injected subcutaneously in athymic mice. Tumor growth was measured daily with a caliper and tumor volume was calculated with the formula: 0.52 × width 2 × length. Tumor growth rates were also determined. Treatment started the cell inoculation day. A clinically relevant dose of dDAVP or [V^4^Q^5^]dDAVP (0.3 µg kg^−1^) was administered intravenously thrice a week (38). Animals in control group received saline vehicle. When control group reached a mean tumor volume of 1,300 mm^3^, animals from all experimental groups were sacrificed.

### Statistical Analysis

Statistical significance was evaluated using Prism 6 statistical software (GraphPad, Inc., CA, USA). Results presented in this study are expressed as mean values ± SEM, mean values ± SD, or mean ± confidence interval (CI). The normal distribution of data was confirmed using the Shapiro–Wilk normality test and the homoscedasticity was determined with Bartlett’s test. For multiple comparisons between experimental groups one-way ANOVA (followed by Dunnett or Tukey posteriori test) or two-way ANOVA (followed by mean 95% CI comparison test) were performed. For repeated measures ANOVA, the Geisser–Greenhouse correction was used for assumption of sphericity.

Doubling time was calculated using the exponential growth equations and the fits were compared. Growth rates represent the slopes of the linear regression of the tumors volumes from day 17 to 25. Data correspond of at least three independent experiments. Significant levels were defined as *p* < 0.05.

## Results

### Expression of V2r in Lung and Prostate Cells

**Figure 1:**
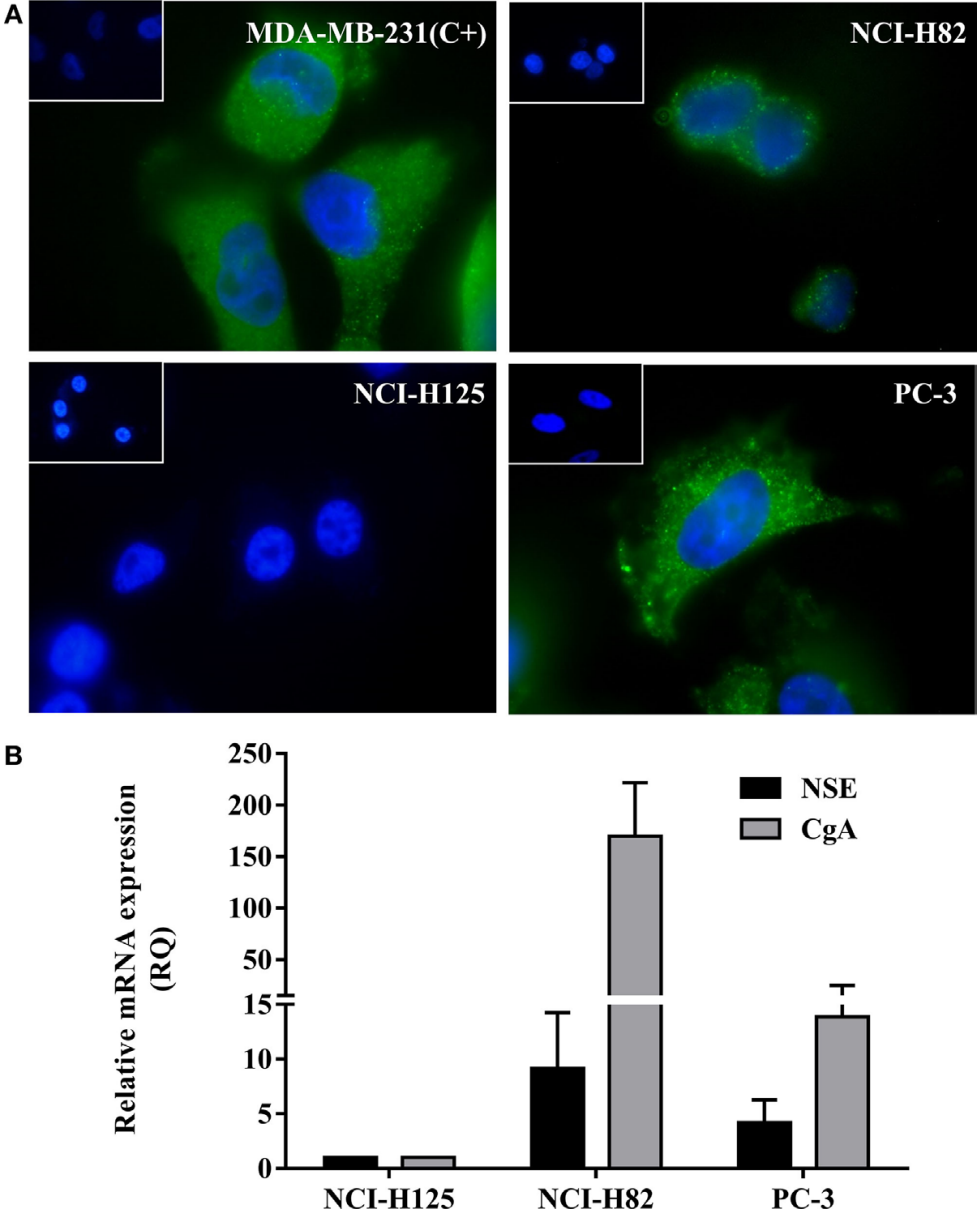
**Expression of V2r by Immunofluorescence detection and of neuroendocrine (NE) markers by quantitative RT-PCR (qRT-PCR)**. **(A)** V2r expression was detected using a specific anti-V2r antibody and a secondary antibody labeled with FITC-conjugated goat anti-rabbit IgG. Nuclei were labeled with DAPI. Negative controls consisted of omission of the primary antibody and were consistently negative (Insets). NCI-H82 human small cell lung cancer cells and PC-3 human prostate cancer cells expressed the V2r. NCI-H125 human nSCLC cells were negative for the receptor. MDA-MB-231 human breast carcinoma cells were used as a positive control (original magnification ×1,000). **(B)** For expression of NE markers, chromogranin A and specific neuronal enolase expression levels were evaluated by qRT-PCR in NCI-H82 and PC-3 cells. Each sample was analyzed in triplicate and mean cycle threshold values (Ct) were used for further analysis. Ct values were normalized for GAPDH expression levels and expressed in relation to control samples (NCI-H125). Relative quantification values were calculated as 2^−ΔΔCt^. Results are expressed as mean ± SEM.

In order to confirm the expression of V2r in tumor cells, an immunofluorescence assay was conducted. As shown in Figure 1A, NCI-H82 and PC-3- cells express the V2r. NCI-H125 human non-small lung cancer cells are negative for V2r. MDA-MB-231, a cell line known to display AVP membrane receptors, was used as a positive control of V2r expression (32).

### Expression of NE Markers in the Tumor Cell Models

Chromogranin A and NSE are the most commonly employed markers to determine NE characteristics, both at the tissue and in general circulation (39). In order to determine NE differentiation, the expression of NE markers, CgA and NSE, was evaluated by qRT-PCR. As shown in Figure 1B, qRT-PCR studies demonstrate that SCLC NCI-H82 and hormone-independent PC-3 cells express NE markers.

### Cytostatic Effect of V2r Stimulation by dDAVP and [V^4^Q^5^]dDAVP in Lung and Prostate Cancer Cells

**Figure 2:**
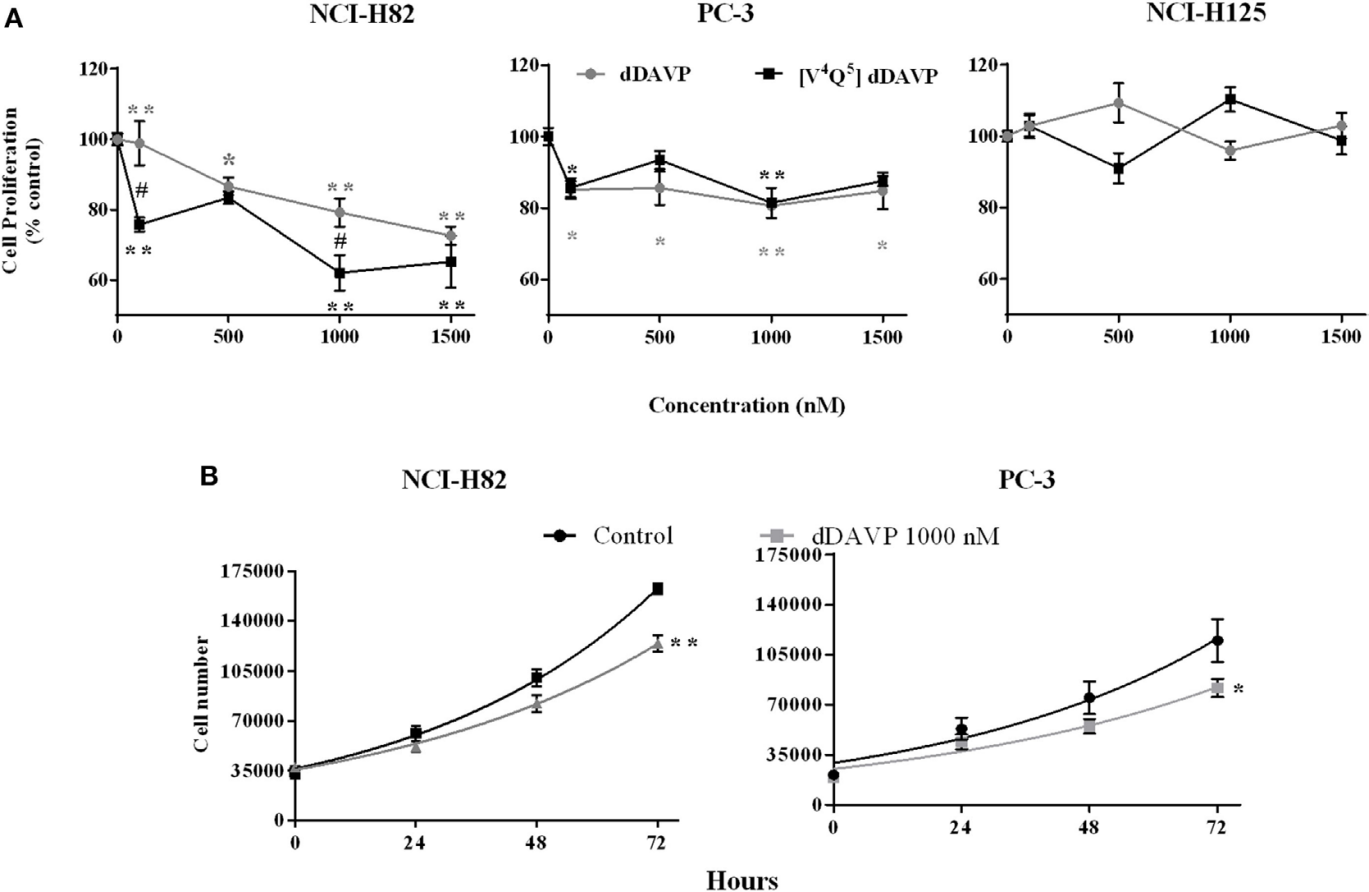
**Antiproliferative effects of vasopressin analogs on lung and prostate cancer cells**. Effect of desmopressin (dDAVP) and [V^4^Q^5^]dDAVP (100–1,000 nM) on log-phase growing cells was measured using the MTS or MTT assays. The optical density of control cells was considered as 100% viability. **(A)** Effect of dDAVP and [V^4^Q^5^]dDAVP (100–1,500 nM) on NCI-H82, PC-3, and NCI-H125 cells. Results are expressed as mean ± 95% confidence interval (CI). Two-way ANOVA followed by mean 95% CI comparison *post hoc*. *p < 0.05; **p < 0.01 (corresponding to each analog concentration vs. its control) ^#^*p* < 0.05 (dDAVP concentration vs. same [V^4^Q^5^]dDAVP concentration). For doubling time calculation; viable cells were counted every 24 h for 72 h using trypan blue dye exclusion. Doubling time with and without dDAVP 1,000 nM treatment was calculated with the exponential phase of growth, control and treatment curves fit at 72 h were compared on each cell line. **(B)** Doubling time of NCI-H82 and PC-3 cells. Results are expressed as mean ± SEM of cells number at 72 h. Comparison of fits **p < 0.001.

We evaluated the cytostatic effect of the parental peptide dDAVP (40) and the analog [V^4^Q^5^]dDAVP on log-phase growing lung and prostate cancer cells. After a 72 h exposure, both peptides significantly reduced proliferation in NCI-H82 and PC-3 tumor cell cultures. Additionally, we confirmed that V2r-negative NCI-H125 cells did not modify their proliferative behavior in response to these peptides (Figure 2A).

In the NCI-H82 cell line, [V^4^Q^5^]dDAVP analog showed a higher cytostatic effect than dDAVP, at high (1,000 nM) as well as low (100 nM) concentrations, reducing cell proliferation by up to 40% (Figure 2A). In the PC-3 cell line, both peptides have similar antiproliferative effects reducing tumor cell growth by up to 15%.

To reconfirm the cytostatic effect of dDAVP on the studied cell lines, we evaluated doubling time with and without treatment (Figure 2B). The doubling time without treatment was 33 and 36 h in NCI-H82 and PC-3, respectively. DDAVP treatment increased the doubling time in both cell lines: 7 h in NCI-H82 and 6 h in PC-3. The comparison of fits was significate in both lines (NCI-H82 control cells doubling time: 33 h, *R* = 0.98; NCI-H82 dDAVP treatment cells doubling time: 40 h, *R* = 0.96; PC-3 control cells doubling time: 36 h, *R* = 0.92; PC-3 dDAVP treatment cells doubling time: 42 h, *R* = 0.93).

In order to complement high density proliferation assays, we studied the peptide ability to modify *in vitro* low density cell growth using a clonogenic growth assay (41).

**Figure 3:**
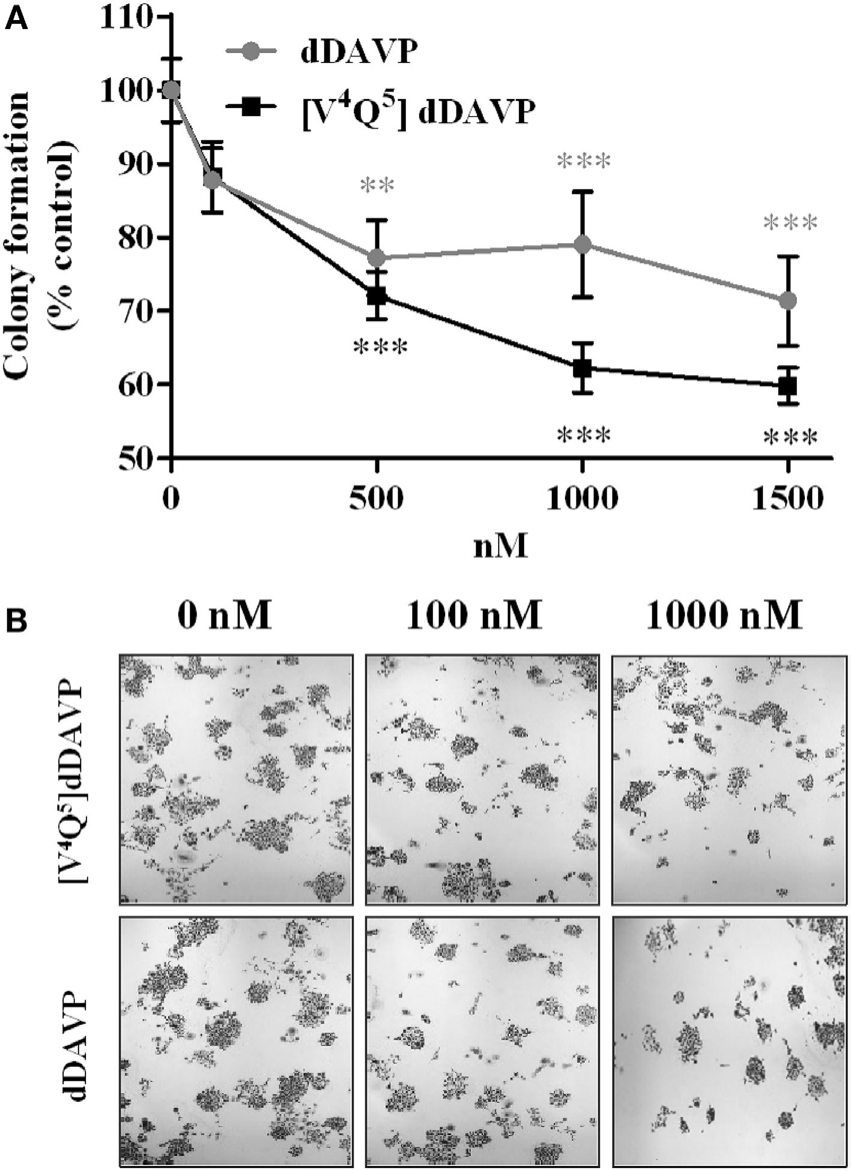
**Effect of vasopressin analogs on clonogenic growth of PC-3 cells**. **(A)** Effect of desmopressin or [V^4^Q^5^]dDAVP treatment on clonogenic growth of PC-3 cells. Cells were seeded in 96-well plates and incubated with varying concentrations of compounds (100–1,500 nM) in complete medium for 7 days. Results are expressed as mean ± confidence interval 95%. Two-way ANOVA proved not significant for the interaction and the factor “analog” and significant for the factor “concentration.” Dunnett *post hoc* test corresponding to each analog concentration vs. its control; *p < 0.05; **p < 0.01. **(B)** Representative photographs of PC-3 colonies at different concentrations (original magnification ×40).

Incubation with dDAVP and [V^4^Q^5^]dDAVP (100–1,500 nM) during 1 week significantly reduced the number of PC-3 tumor cell colonies showing a higher effect than the observed in proliferation assays using log-phase growing cells, inhibiting clonogenic growth by up to 30 or 40%, respectively (Figure 3).

### Silencing of V2r on Tumor Cell Lines

**Figure 4:**
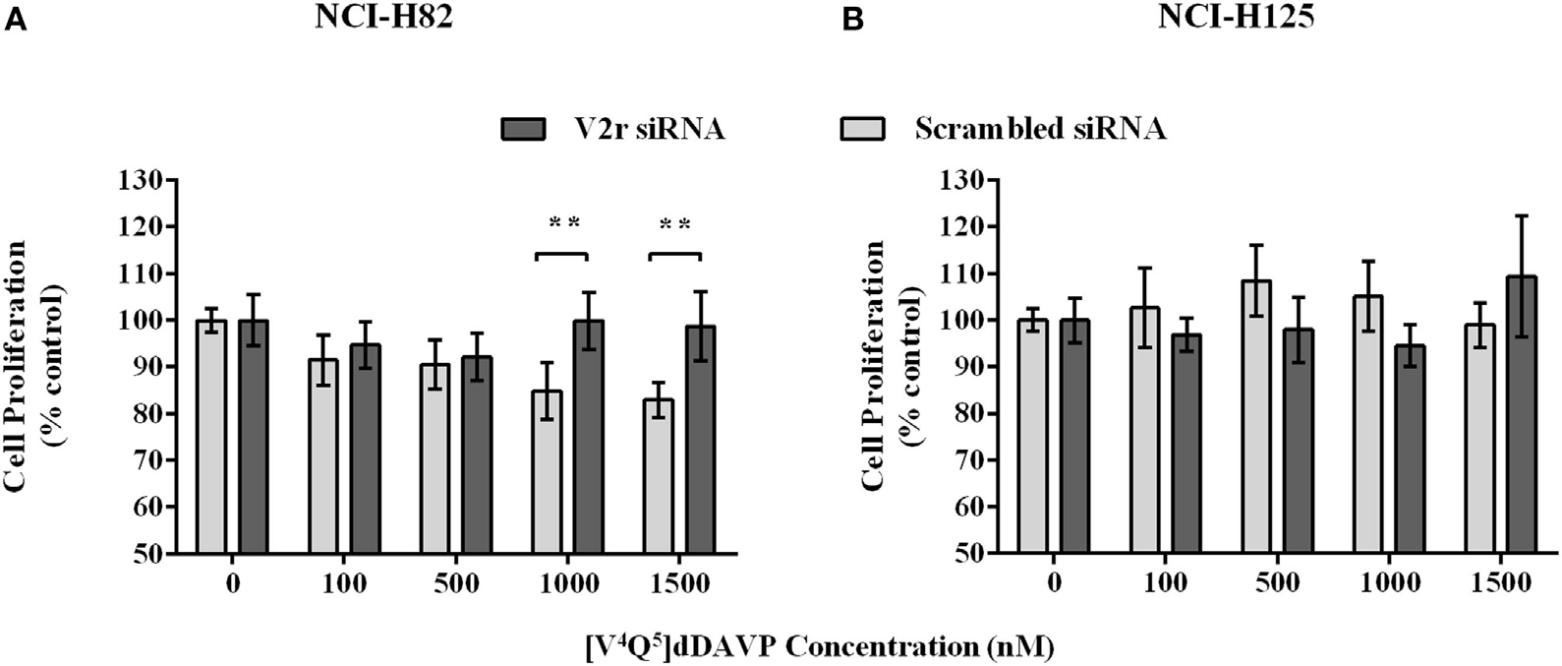
**Silencing of V2r on NCI-H82 and PC-3 cells**. NCI-H82 and NCI-H125 cells were transfected with V2r small interfering RNA (siRNA) or control siRNA using Lipofectamine 2000. On day 7, cells were plated in 96-well plates and then treated with different concentrations (100–1,500 nM) of [V^4^Q^5^]dDAVP for 72 h. Cell growth was measured by colorimetric MTT or MTS assay. **(A)** Effect of [V^4^Q^5^]dDAVP on V2r silenced NCI-H82 cells. **(B)** Effect of [V^4^Q^5^]dDAVP on V2r silenced NCI-H125 cells. Results are expressed as mean ± confidence interval 95%. Two-way ANOVA followed by mean 95% confidence interval comparison *post hoc* **p < 0.01.

Additionally, to determined that [V^4^Q^5^]dDAVP acts by V2r, we interfered the expression of the molecule. V2r was depleted using siRNA on NCI-H82 and NCI-H125 cells. The silencing of V2r significantly attenuated the inhibitory effects of [V^4^Q^5^]dDAVP on cell proliferation compared to control siRNA-transfected NCI-H82 cells. The silencing had no effect on NCI-H125 (Figure 4), indicating that antiproliferative effect mainly results from V2r activation.

### Effect of dDAVP and [V^4^Q^5^]dDAVP on Tumor Cell Motility

**Figure 5:**
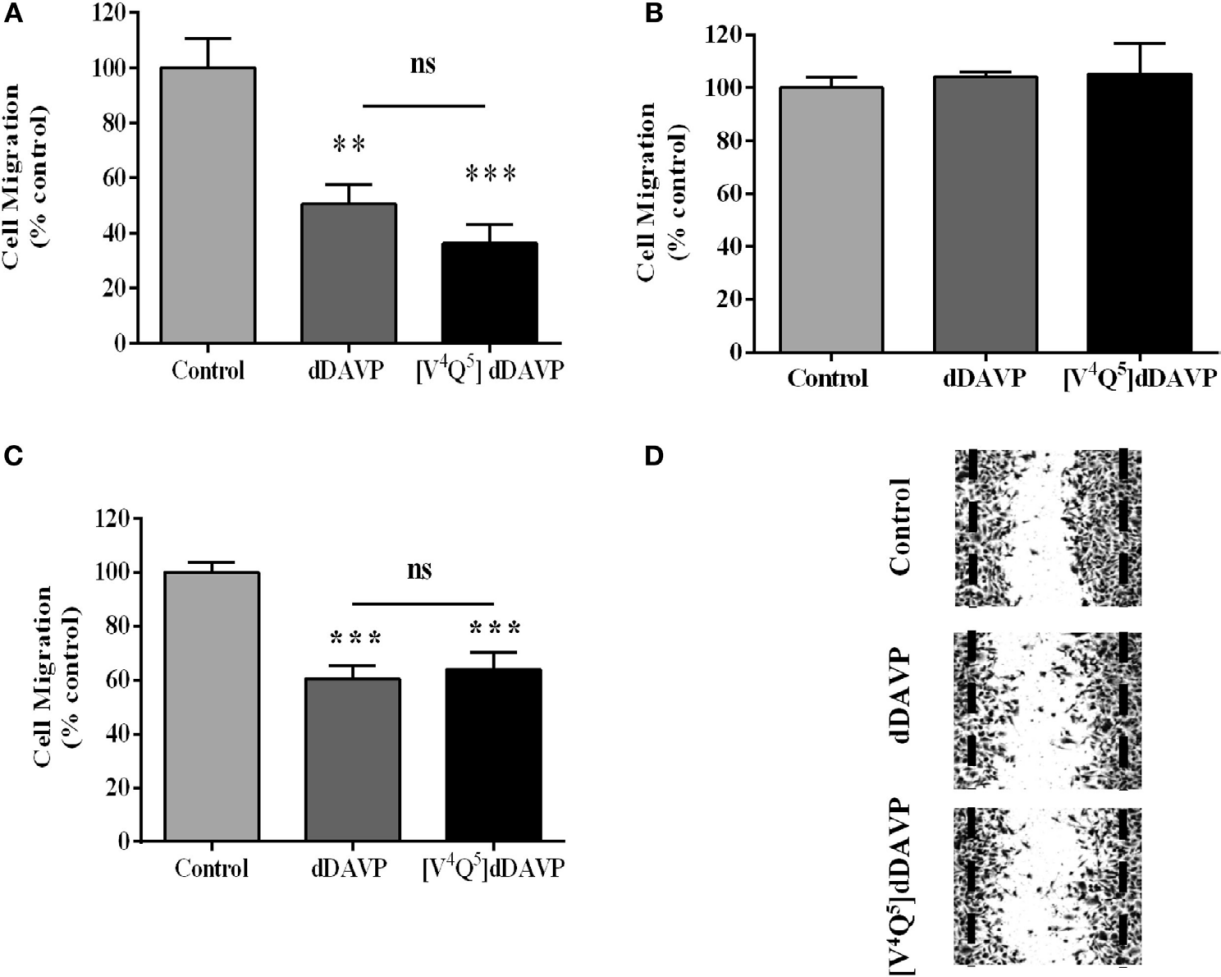
**Effect of desmopressin (dDAVP) and [V^4^Q^5^]dDAVP on lung and prostate cancer cells migration**. NCI-H82 cells or NCI-H125 cells were starved and were added to the top chambers of 24-well Transwell^®^ permeable support with 8 µm pore in serum-free medium. The lower chamber was filled with medium containing 10% fetal bovine serum and dDAVP or [V^4^Q^5^]dDAVP at a final concentration of 1,000 nM. After incubating for 24 h, migrated cells on the bottom of the insert were stained with crystal violet (NCI-H125) or counted in Neubauer chamber (NCI-H82 cells). The number of migrating cells randomly selected in five fields was counted. Migration of PC-3 cells was measured using wound-healing assay. Wounds were created by scraping confluent PC-3 monolayers. After 16 h incubation in the presence or absence of dDAVP or [V^4^Q^5^]dDAVP at a final concentration of 1,000 nM, cells were fixed and stained. Wounds were quantified using Image J. software. Wound closure measurements were normalized to the maximum scratch area. **(A)** Effect of dDAVP and [V^4^Q^5^]dDAVP on Transwell^®^ migration of NCI-H82. **(B)** Effect of dDAVP and [V^4^Q^5^]dDAVP on Transwell^®^ migration of NCI-H125. **(C)** Effect of dDAVP and [V^4^Q^5^]dDAVP on wound migration of PC-3. **(D)** Representative microphotographs of PC-3 cells wound migration. Results are expressed as mean ± SEM One-way ANOVA followed by Tukey *post hoc* test. **p < 0.01; ***p < 0.001.

*In vitro* treatment at 1,000 nM concentration of both studied peptidic analogs decreased tumor cell migration by 60–70% in NCI-H82 (Figure 5A) and 30–40% in PC3 (Figures 5C,D), tumor cells using Transwell^®^ and wound assays, respectively. DDAVP and [V^4^Q^5^]dDAVP did not affect migratory capacity of V2r-negative NCI-H125 cells (Figure 5B).

### Regulation of NE Markers in Tumor Cells by dDAVP Treatment

**Figure 6:**
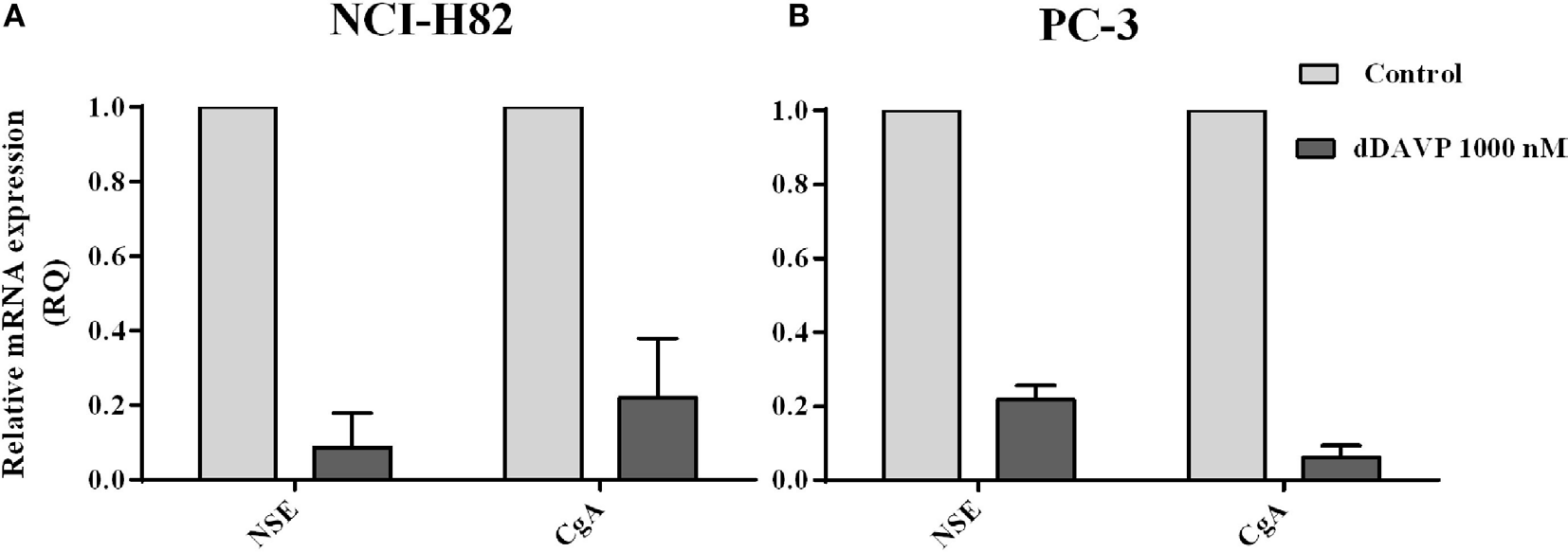
**Modulation of neuroendocrine markers in lung and prostate cancer cells by desmopressin (dDAVP) treatment**. The relative mRNA expression of specific neuronal enolase (NSE) and chromogranin A (CgA) was evaluated by quantitative RT-PCR in NCI-H82 and PC-3 cells incubated with 1,000 nM of dDAVP as described in the Figure 1. Quantification relative CgA and NSE in **(A)** NCI H82 cells and **(B)** PC-3 cells.

As previously shown, NCI-H82 and PC-3 cells express high levels of NE markers with clinical relevance. In order to evaluate if dDAVP by V2r has the capacity of modulating NE markers level expression, transcript levels in tumor cells were measures by qRT-PCR after ON incubation with 1,000 nM of dDAVP. As shown in Figure 6, the expression of NE markers was dramatically reduced after treatment by 90% for NSE and 80% for CgA on NCI-H82 cells and by 80% for NSE and 95% for CgA on PC-3 cells (Figure 6).

### Effect of dDAVP and [V^4^Q^5^]dDAVP on Xenograft Tumor Progression

**Figure 7:**
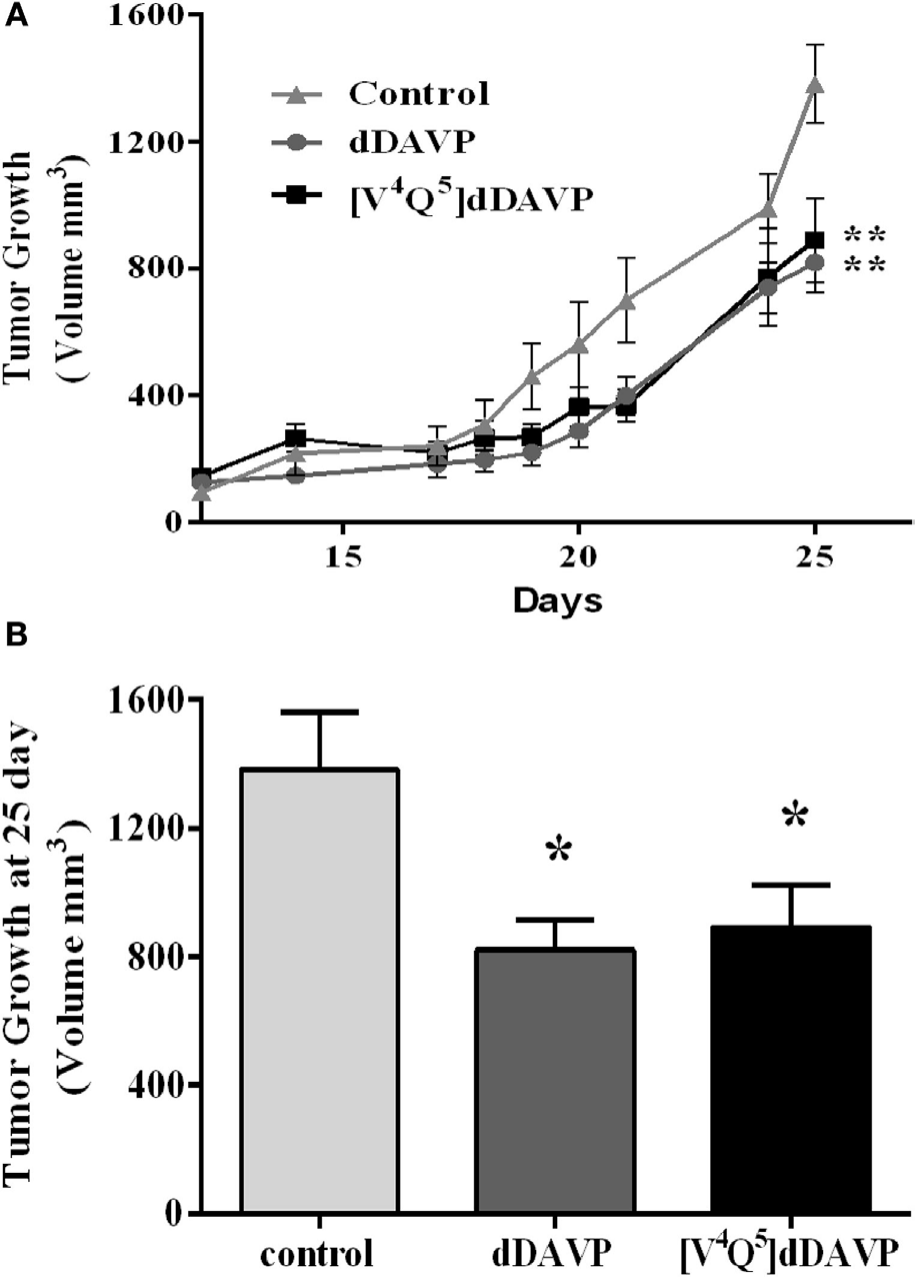
**Effect of treatment of desmopressin (dDAVP) and [V^4^Q^5^]dDAVP on tumor growth of lung cancer xenografts**. **(A)** NCI-H82 tumor volume in mice receiving saline vehicle (control) or 0.3 μg kg^−1^ of dDAVP or [V^4^Q^5^]dDAVP over time. To generate lung cancer xenografts, NCI-H82 cells and Matrigel^®^ were injected subcutaneously in athymic mice. Tumors were measured daily with a caliper and tumor volume was calculated. dDAVP or [V^4^Q^5^]dDAVP were administered intravenously thrice a week. When the control group reached a mean tumor volume of 1,300 mm^3^, animals were sacrificed. Results are expressed as mean ± SEM Repeated measures ANOVA test followed by Tukey *post hoc* test ***p < 0.001. **(B)** Comparison of tumor volume at day 25. Results are expressed as mean ± SD. One-way ANOVA followed by Dunnett post (**p < 0.01; ***p < 0.001).

Finally, we explored the effect of dDAVP and [V^4^Q^5^]dDAVP in NCI-H82 xenograft growth in nude mice. Briefly, mice received thrice a week intravenous doses of the analogs (0.3 µg kg^−1^) after tumor confirmation by palpation. Treatment for 25 days with both peptide analogs significantly reduced final tumor volume by 40% in comparison vehicle-treated group (Figures 7A,B). Tumor growth rates (from day 17 to 25) were also reduced by about 35% after dDAVP or [V^4^Q^5^]dDAVP treatment (rates growth: control:132.4 ± 14.64; dDAVP: 86.8 ± 8.37; [V^4^Q^5^]dDAVP: 86.54 ± 11.73). Rates growth expressed as slope ± SD.

## Discussion

The participation of neuropeptides in tumor growth, particularly in NETs, has always been object of study with the aim of discovering mechanisms involved in cancer progression and new therapeutic targets. NETs are a heterogeneous group of tumors with different characteristics. These tumors are, generally, aggressive and resistant to current therapies. Although NETs are rare, these have been increasing in incidence. Symptoms are often non-specific and do not necessarily lead to the identification of the specific lend themselves to identifying the specific underlying tumor. The NETs cells were characterized by the expression of markers such as neuropeptides. These circulating markers from specific tumor types can be used for diagnostic and prognostic information of NETs (40). The expression of AVP receptors on several types of tumors like SCLC and breast cancer highlights the multifaceted role of AVP, such as predicted by North et al. (17–20). Additionally, it was recently reported that different AVP receptors are expressed throughout the gastrointestinal tract tumors, including colon and pancreas, with interesting pathophysiological implications (14, 15). Unlike V1ar and V1br, V2r is associated with antiproliferative signaling, involving activation of adenylate cyclase followed by intracellular cAMP elevation (41). Increases in cAMP levels using cAMP analogs or cAMP elevating agents such as forskolin or different hormone derivatives can trigger cell cycle arrest at G1 phase and induce apoptosis in numerous cancer cell types, suppressing tumor growth. These antiproliferative effects are mediated by PKA and correlated with the increase of cell cycle inhibitor p27^kip1^ and the decrease of antiapopotic bcl2 protein levels in MDA-MB-231 cells. Proliferative signal transmission through the Ras/Raf/ERK pathway is blocked by the elevation of cellular cAMP levels mainly *via* PKA-mediated Raf inhibition (42, 43). We have demonstrated that the treatment with [V^4^Q^5^]dDAVP resulted in partial arrest of MDA-MB-231 cells in G0/G1 phase (32), further studies on apoptosis are needed.

Because stimulation of this pathway can trigger deleterious effects on cancer cells (23), compound like dDAVP which specifically activate V2r, have a potential role as anticancer agents (44, 45). dDAVP has demonstrated to display antitumor properties in breast and colorectal cancer, showing an angiostatic and antimetastatic effect, disrupting cooperative interactions of tumor and endothelial cells during tumor progression (28, 30).

[V^4^Q^5^]dDAVP was selected from a panel of peptidic analogs derivatized from dDAVP with different structural modifications (31). Novel compound [V^4^Q^5^]dDAVP exhibited a significantly higher cytostatic, angiostatic, and antimetastatic effects against breast cancer cells than the parental compound (32, 33).

In this work, we examined the impact of selective stimulation of V2r on key cellular events related to tumor progression through the use of the two analogs V2r agonists, dDAVP and [V^4^Q^5^]dDAVP, of the neuropeptide AVP, on lung and prostate tumor lines with NE phenotypes. NCI-H82 and PC-3 cancer cells are highly aggressive human cell lines which, as we show in the present study, express high levels of NE markers NSE and CgA as well as the AVP receptor V2. DDAVP and [V^4^Q^5^]dDAVP significantly reduced proliferation, doubling time, and migration in both tumor cell cultures. [V4Q5]dDAVP analog showed a higher cytostatic effect than dDAVP, both at low and high concentrations, obtaining a maximum inhibition of 40% on cellular proliferation in the NCI-H82 cell line. As early exposed, activation of V2r signaling involves an increase in cAMP levels. It has been described that stimulation of cAMP/PKA pathway result in inhibition of cell migration in different cancer cell (46, 47). In lung cancer cells, migration inhibition was also greater with [V^4^Q^5^]dDAVP than with parental peptide reducing cell motility by 64 and 50%, respectively, although this difference was not statistically significant.

Antitumor profile of dDAVP on PC-3 cells was similar to that obtained in a study by Sasaki et al., where they reported the antiproliferative and anti-invasive ability of dDAVP on prostate cancer cells related to antiproliferative and antimigratory ability (48). Interestingly, [V^4^Q^5^]dDAVP was not better than dDAVP in the PC-3 model and this difference could be related to the characteristics of the cell line.

It has been reported that, under certain culture conditions, only some subpopulation of PC-3 cells undergo NE transdifferentiation. In this highly heterogeneous cell line, it may be possible that only a fraction of the total cell population express NE markers or V2r. In the latter, inhibition of cell growth by V2r agonists may be subtle and differences between analogs may be more difficult to assess (49).

For this reason, we, additionally, studied peptides ability to modify *in vitro* low density cell growth using a clonogenic growth assay. This method complemented MTT assay, because the conditions are most limiting, and thus, this assay is better for the evaluation of new anticancer agents (50). The inhibitory effect of both analogs was higher than the observed in proliferation assays using log-phase growing cells and [V^4^Q^5^]dDAVP analog showed a major cytostatic effect than dDAVP inhibiting up to 40% of cell growth at high concentrations, although this difference was not statistically significate.

We also showed that [V^4^Q^5^]dDAVP acts *via* V2r. The silencing of V2r significantly attenuated the inhibitory effects of [V^4^Q^5^]dDAVP on cell proliferation. This result complements the already reported findings by our group, where chemical V2r blockade by the selective antagonist tolvaptan completely abolished [V^4^Q^5^]dDAVP effects in MDA-MB-231 breast cancer cells (32) and, also, the findings by Keegan et al. (23), where the use of satavaptan (another non-peptidic V2r antagonist) blocked the mild cytostatic effects of dDAVP on human breast cancer cells.

Neuroendocrine differentiation of tumors is related with the transition from cancer cells with epithelial features to cells with invasive and metastatic phenotypes (51). Interestingly, after dDAVP treatment, expression of both NE markers studied was reduced on lung and prostate cancer cell lines. In prostate cancer, transition from primarily androgen-dependent tumor toward a hormone-independent tumor represents an unfavorable prognosis. NE transdifferentiation is fundamental in this process and contributes to tumor progression and hormone resistance (52, 53). Additionally, in lung cancer, transformation from nSCLC to SCLC was associated with poor treatment response and rise of NSE in serum (8, 54). A reduction of the expression of NE markers could indicate NE dedifferentiation. In cancer patients, elevated CgA and NSE levels correlates with tumor burden, number of dissemination sites, and lack of clinical response, while a decrease of these biomarkers might indicate a better prognosis (55). dDAVP capacity to modulate NE markers in different tumor types constitutes an interesting and important contribution for further studies.

Finally, we explored the *in vivo* effect of dDAVP and [V^4^Q^5^]dDAVP on NCI-H82 SCLC xenografts. Intravenous injection of clinically relevant doses of both analogs provoked an important reduction of subcutaneous tumor volume compared to control group. According to the *in vitro* effect observed, treated tumors grew at a 35% slower rate in comparison to vehicle-treated animals. In these experimental conditions, [V^4^Q^5^]dDAVP analog did not show a higher effect than the parental peptide dDAVP. [V^4^Q^5^]dDAVP analog has displayed a more potent activity *in vivo* in several models of breast cancer compared to parental drug. There is a probability that aggressive tumors as SCLC with an extremely fast expansion and rate of growth demand a higher dose to show differences between compounds. These results are preliminaries and require further studies *in vivo*, mainly, of dose evaluation to conclude.

NCI-H82 represents a valuable disease model of recurrent and drug-resistant NE SCLC (8). This is the first time that the efficacy of these synthetic analogs of AVP as antitumor agents on SCLC is demonstrated. Nowadays, targeted therapies like interferon, SSA, and VEGF and mTOR inhibitors became an integral part of therapies for NE tumors, particularly for well-differentiated and slow-growing gastroenteropancreatic tumors while chemotherapy is reserved for poorly differentiated and progressive tumors. Successful treatment of disseminated NETs requires a multimodal approach where radical tumor surgery may be curative but is rarely possible (56).

Selective compounds like dDAVP and [V^4^Q^5^]dDAVP, which trigger specific biological actions through V2r, modulating tumor growth with low toxicity represent interesting candidates as antitumors agents.

In this work, we demonstrated that the specific agonists of V2r, dDAVP, and [V^4^Q^5^]dDAVP display antitumor capacity on different human models of lung and prostate cancers with NE features. Elucidation of the mechanism involved in the signaling pathway of V2r is not complete, but further investigation is needed. Our findings show the potential benefit of these compounds on aggressive tumors NE phenotypes.

## Author Contributions

MP, DA, and GR contributed to the conception and design of research; MP, JG, CC, and NG performed experiments; MP, JG, DA, and GR acquired, analyzed, and interpreted the data; MP prepared figures; MP and GR participated in the writing and revision of the manuscript; MP, JG, DA, and GR drafted, edited, revised critically, and approved final version of manuscript. All authors read and approved the final version of manuscript.

## Conflict of Interest Statement

The authors declare that the research was conducted in the absence of any commercial or financial relationships that could be construed as a potential conflict of interest. The reviewer GR and handling Editor declared their shared affiliation, and the handling Editor states that the process nevertheless met the standards of a fair and objective review.
